# Supplementary material for: A qualitative study on factors influencing health workers’ uptake of a pilot surgical antibiotic prophylaxis stewardship programme in selected Georgian hospitals
Source: PLOS Glob Public Health. 2025 Apr 16;5(4):e0003493. doi: 10.1371/journal.pgph.0003493 (PMC12002478; doi:10.1371/journal.pgph.0003493)
Supplement: S1 Data — (DOCX) [file pgph.0003493.s002.docx]

**Georgia TAP AMS Project – COM-B Findings for Thematic Analysis**

|  | **CAPABILITY** | **OPPORTUNITY** | **MOTIVATION** | **BEHAVIOUR** |
| --- | --- | --- | --- | --- |
| **Administrators** | - Non-AMS administrators lack information on AMS programmes. Their functions do not include supervising the use of antibiotics for prophylactic or curative purposes in the process of protecting patients from infectious diseases. - AMS administrators are involved in the establishment of protocols for the Rational Antimicrobial Stewardship Program. This includes the development of guidelines for the rational use of antibiotics, their implementation in the work process and the measurement of the effectiveness of the use of targeted antimicrobial drug management techniques in practice through pre-developed indicators. | - Non-AMS administrators use MoH protocols and in one hospital they operated based on experience. - One hospital experiences AB shortages, but this is beyond administrators' remit and handled by financial department | - Epidemiologists believe administrators can motivate staff to accept and adhere to AMS | - Non-AMS administrators do not participate in the preparation/modification of the protocols of the rational management of antimicrobial medicines, as they are guided by the protocol provided by the Ministry of Health. - Administrators who are also clinical managers in AMS hospitals monitor patient treatment including AB prescription - By participating in ICARS project, the professional competences of epidemiologists improved significantly. - People with administrative positions note that the monitoring of the use of antibiotics in clinics is actively and strictly carried out. |

|  | **CAPABILITY** | **OPPORTUNITY** | **MOTIVATION** | **BEHAVIOUR** |
| --- | --- | --- | --- | --- |
| **Epidemiologists** | - Epidemiologists representing both groups of hospitals conduct bacteriological analyzes and determination of sensitivity to antibiotics, as well as the study of allergic reactions to antibiotics. - In non AMS hospitals they experience a lack of information about the antibiotic prophylaxis program during surgical interventions and think that it is important for them to conduct qualification-raising trainings. - AMS-hospital epidemiologists are aware of AWaRe classification, but not non-AMS epidemiologists - In AMS hospitals they administer the professional training of the middle and low-level employees of the medical institution and teach them new approaches/methods - In AMS hospitals they actively participate in the process of rational use of antibiotics (for example, they monitor the compliance of the patient's diagnosis and the antimicrobial drug prescribed by the attending physician, check how correctly the dose and time of administration of the antibiotic was determined, etc.) | - Educational events in non AMS hospitals regarding the rational management of antibiotics were not conducted directly for epidemiologists. - In AMS hospitals, they report participating in various trainings, seminars and conferences in order to raise their qualification, but not in non-AMS hospitals (only 1 person). - Long-term legislative integration of AMS needed for sustainability - Long-term sustainability depends on funding for lab tests as well | - In non AMS hospitals they are involved in the process of selecting optimal antibiotics for treatment. However, they believe that they should also actively participate in making decisions regarding this issue. - They believe their involvement in AB decision is necessary to verify correct AB selection, determine if selected by guidelines, suitability, dosage, etc. | - In AMS hospitals, they participate in AB selection process but not in non-AMS hospitals. - In AMS hospitals epidemiologists provide constant supervision and give instructions to the medical staff directly involved in the patient's treatment process. The instructions refer to such issues as: which antibiotic to use and in what dose, in which cases it should be used in the form of injection and/or pill, etc. - The use of the AWaRe classification system is not discussed by the epidemiologists of the hospitals where the implementation of the ICARS program has not yet started. - AMS-hospital epidemiologists use the AWaRe classification system of antibiotics, which involves differentiating antibiotics into "Watch" and "Reserve" categories. Internal protocols of medical institutions are based on this classification. - Medical institutions involved in the Antimicrobial Resistance Program (AMR) indicate that they periodically send the dynamics of nosocomial infections and antibiotic consumption statistics to the National Center for Disease Control and Public Health. - Those medical institutions where the implementation of the Antimicrobial Resistance Program (AMR) has not yet started indicate that the National Center for Disease Control and Public Health has requested statistical information from their institutions, although such practice does not take the form of periodic reporting. |

|  | **CAPABILITY** | **OPPORTUNITY** | **MOTIVATION** | **BEHAVIOUR** |
| --- | --- | --- | --- | --- |
| **Surgeons** | - Believe AMS programme is important in Georgia - Aware of which AB to use for surgery - Non-AMS surgeon reports an incorrect understanding of resistance | - Both AMS and non-AMS surgeons state medical staff directly involved in the treatment do not have information on when and in what dose antibiotics can be included in SSI. - AMS has strengthened stewardship in the hospital Some non AMS-surgeons say they don't have guidelines for AB use - AMS has increased workload for others - Previous guidelines were also sufficient - Surgeons indicate due to the list of approved antibiotics, doctors can only prescribe expensive state-approved antibiotics that often patients cannot afford. - Resistance from patients who demand AB - Since they have the right to use only antibiotics prescribed and recommended by the Ministry of Health, Non AMS surgeons do not have a wide choice in terms of the use of antibiotics in the process of treating patients. Therefore, they do not work or rarely work on the development of antibiotic treatment tactics. - Surgeons representing both groups state that part of the medical staff of their clinics (who are directly involved in the treatment of patients) do not have information on when and in what dose antibiotics can be included in the treatment of surgical site infection. - In non AMS hospitals there is no internal protocol or guideline for the rational use/management of antibiotics in a medical institution. Only antibiotic medicines determined by the Ministry of Health are used - The use of any antibiotics is recorded - both using an online program and such information is included in the patient's treatment history. According to surgeons, it is better to additionally create special reports, where all the details will be analyzed that will justify the use of specific antibiotics and, at the same time, reveal the gaps observed in the course of treatment. | - Surgeons of clinics not included in the AMR program believe that it is permissible to participate in meetings organized by pharmaceutical companies that deal with the dissemination of information about new drugs. - Some of the surgeons of the clinics involved in the AMR program categorically refuse to participate in any activities organized by pharmaceutical companies, while some believe that participation in meetings and presentations organized by pharmaceutical companies in order to receive information about new drugs is permissible. | - The duties of surgeons of both groups of hospitals include: emergency and planned surgical services for patients, post-operative supervision and treatment. Within the scope of their activities, they have active communication with reanimatologists, anesthesiologists, other surgeons and epidemiologists. - AMS surgeons, along with other specialists, work to develop antibiotic treatment strategies for patients with specific diagnoses. - Since the initiation of the ICARS program, disease-specific protocols have been adopted to provide information/guidelines for rational antibiotic management. |

|  | **CAPABILITY** | **OPPORTUNITY** | **MOTIVATION** | **BEHAVIOUR** |
| --- | --- | --- | --- | --- |
| **Nurses** | - Nurses want more training on AMS to have stronger theoretical knowledge and to communicate with patients - Surgeons believe nurses are not qualified and need training to increase competencies - Some nurses believe that pharmaceutical companies increase competition and development of better drugs. - AMS nurses' responsibilities include communicating with the patient, providing information about the AMR program, contacting the patient at intervals after surgery, and interviewing the patient in order to make a clinical assessment of complaints/complications, as well as collecting information about the antimicrobials that the patient is taking by doctor's prescription or they used it by themselves after being discharged from the clinic, during the treatment process. The assistant chief nurse at a clinic participating in the ICARS program claims to be responsible for overseeing antibiotic therapy. - Non AMS Nurses' responsibilities do not include providing information to patients about rational antibiotic treatment, contacting the patient at intervals after surgery, and asking about health status for clinical evaluation of complaints/complications. Also, they do not participate in collecting information about the antimicrobial agents that were used in the patient's treatment process. - Non-AMS nurses say they don't have enough information on the AMS programme | - Non AMS hospital nurses did not undergo training regarding the rational management of antibiotics during the treatment process. - AMS nurses talked about having opportunities for continuing/continuing education, but non-AMS nurses did not - Due to the fact that antibiotics are not administered rationally in the clinic, timely inclusion of nurses in the AMR program is considered very important. - Nurses representing both groups of hospitals say that the pharmacy of the clinic is responsible for the purchase of antibiotics. Their work experience shows that after the attending physician prescribes an antibiotic, the nurse addresses the clinic's pharmacy with a request for a specific antibiotic. Here, the nurses state that there was no problem of shortage of antimicrobial drugs in their practice. - A certain part of the nurses representing both groups of hospitals do not have information regarding the visit to the clinics of pharmaceutical companies; Unlike other medical personnel, they consider cooperation with pharmaceutical companies in a relatively positive light and say that it is their right to advertise a particular medicine. In addition, they add that the activity of such organizations contributes to the growth of competition in the market, which, ultimately, is a prerequisite for the production of better quality medicines. | - AMS nurses feel their professional competencies have increased through the AMS programme - AMS nurses indicate their workload has not increased significantly. They believe that the additional functions and duties they have within the framework of AMR/SAP are very interesting and important for professional advancement. | - Duties of nurses depend on their positions - AMS nurses communicate with patients, provide info on the programme, follow up after surgery to collect information on ABs used (during/after being in hospital and prescribed by doctor or additionally). - Nurses involved in the Antimicrobial Resistance Program (AMR) say they communicate with patients and provide information about the AMR program research. The nurse records the patient's data and condition and also agrees to follow up with them twice more, 1 week after surgery and 1 month after surgery. The collected information is stored both in the form of a questionnaire (physically) and electronically. - After the patient's discharge from the clinic, on the basis of telephone communication with them, they do not fill in the so-called "Checklists" in which the process of postoperative rehabilitation of the patient will be described in detail (general state of health, use of antibiotics, doses, time, reasons, etc.). |

|  | **CAPABILITY** | **OPPORTUNITY** | **MOTIVATION** | **BEHAVIOUR** |
| --- | --- | --- | --- | --- |
| **AMR champions** |  | - In institutions involved in the AMS program, communication about this program was established with the residency department and training was provided to the traumatology and surgery departments. The program of rational use of antibiotics was presented to all services. A number of trainings and conferences about SAP are held, where anyone can attend. - It is important to create protocols to promote the rational use of antibiotics, which clinics will use en masse and take into account in the treatment process. In addition, it was noted that the protocol should be understandable not only to the representatives of the medical field, but also to the patients themselves, so that they feel safe during the treatment process and do not look skeptically at the prescription by the doctor. - Some of the facilities where the AMR program has not yet been implemented, did not have pre-developed or research-based guidelines/methods for antibiotic treatment and were guided only by their own experience. - In those clinics, where the implementation of the AMR program has not been started, training within the framework of the program of rational use of antibiotics has not yet been conducted. Similar experiences of program content are shared among staff. - The role of AMR champions representing both groups of clinicians in terms of prescribing/using antimicrobial agents in the treatment process depends on their clinical profile. Some of them hold the position of general surgeon, and some are the head of the surgical department. Thus, in addition to the fact that they have direct communication with patients, they are more or less involved in the administrative activities of the clinic, which means leading various departments, communicating with doctors, supervising and monitoring their activities, monitoring the condition of patients and preparing various clinical documents. As the respondents pointed out, prescribing an antibiotic for a patient is the prerogative of his attending physician. Thus, they are involved in the process only when there is a need for it in the clinic (eg, when the attending physician cannot make a decision independently and a council is needed). Here, they state that the process of starting treatment with antimicrobial agents is preceded by a laboratory study to determine the sensitivity of pathogenic microbes to this or that antibiotic (antibiogram), on the basis of which the optimal medicine is selected by mutual agreement. - According to AMR champions, the administration will issue instructions on the rational management of antimicrobial drugs in both groups of clinics. Also, in the process of selecting an antibiotic, informal consultations are held among colleagues, which helps to determine the appropriateness of using this or that medicine. - Any medication spent/used in the clinics is recorded and registered in the prescription program. - As for the control of the stocks in the clinics, as the AMR champions mentioned, representatives of the pharmacy of the clinic monitor the stock of medicines and, in case of shortage of a particular drug, ensure its timely purchase. | - AMR champions claim that there is a mismanagement of antibiotic treatment in Georgia, which leads to irrational use of antimicrobial drugs in the treatment process. - Initial negative attitude of staff toward AMS programme and increased workloads, but this was resolved | - Role in AMS determined by position. - Majority of champions participate patient treatment. Some champions are involved in administrative activities of the clinic (eg communicating with, supervising, and monitoring activities of doctors, monitoring patients condition, preparing clinical documents, etc - AMR Champions are involved in AB prescription decision making only when attending physician cannot independently choose strategy - AMR champions claim that the practice of rational antibiotic use in their clinics existed even before the ICARS program began. The decision to use antimicrobial drugs, which combines the appropriate dosage of the drug with the diagnosis, appointment interval and form, was determined by the type of operation and the patient's general health condition. |

|  | **CAPABILITY** | **OPPORTUNITY** | **MOTIVATION** | **BEHAVIOUR** |
| --- | --- | --- | --- | --- |
| **Hospital Pharmacist** | - They play an important role in providing complete information to doctors about the pros and cons of using a particular antibiotic. - They report as a result of the control of antimicrobial drugs, the rates of use of these drugs in the treatment process have significantly decreased | - On a monthly basis, they communicate with ICARS representatives and hand over the cards filled by the clinicians about surgical site infections, current prevalence studies, antibiotics used and other important issues.   Clinical pharmacist reports that pharmaceutical company representatives make clinic visits, but are not personally involved in communication with them. |  | - The duties of clinical pharmacists are associated with controlling the use of medicines in the hospital. In particular, they choose cost-effective medicines, monitor the medicines prescribed in case of specific diseases, their mutual compatibility, control the frequency and needs of antimicrobial medicines. - According to pharmacists, there were standards in the clinics that determined which antibiotic should be used at what time. After the clinic joined the ICARS program, the mentioned standards were modified and adapted to international requirements of rational management of antibiotics. In addition, respondents state their institutions use the AWaRe classification system for antimicrobials, as the basis for management of drugs. |

|  | **CAPABILITY** | **OPPORTUNITY** | **MOTIVATION** | **BEHAVIOUR** |
| --- | --- | --- | --- | --- |
| **Professional Association Representatives** | - The association is often approached with questions indicating lack of information among health workers - Assoc reps for epidemiology/surgeons both think HCW in regions have lower access to up-to-date information - representative of the Association of Epidemiologists notes that the majority of clinics do not adhere to the guideline, which recommends the administration of prophylactic antimicrobial drug at a certain point right before surgery, and instead, they start intervention a few days earlier. - Epidemiologists note sustainability of AMR depends on reminding HCW of appropriate AB use after AMS has ended | - Association of Surgeons states that one of the main challenges is following the mandatory guidelines issued by the Ministry of Health without questioning them - Association of Epidemiologists can train medical facility staff on various issues, but little interest; hospitals are willing only if provided for free |  | - Association of Epidemiologists developed a CME programme on infection prevention and control in collaboration with Tbilisi State Medical University, Since one-time training course is not sufficient, they organize additional free courses. - Association of Professionals in Epidemiology cooperate with MOH on regulations and guidelines, provide healthcare facilities with information on govt decisions and legislative changes to ensure implementation in clinics. They carry out projects with the WHO - Association of Surgeons states that they organize visits to regions, hold information meetings with doctors and patients in order to provide Association members with new information and practices. They also offer online consultations, organize conferences on medical issues, and participate in international webinars |

|  | **CAPABILITY** | **OPPORTUNITY** | **MOTIVATION** | **BEHAVIOUR** |
| --- | --- | --- | --- | --- |
| **Pharmaceutical Company Representatives** | - Pharmaceutical company representative thinks broad-spectrum AB are optimal treatment means as they solve health issues in a short time and speed up recovery |  | - The respondent also states that he/she is not interested in the AMR programme currently but can obtain information if interested | - Administrators see companies as suggesting AB as a solution to problems, rather than suggesting alternatives. |
