# Supplementary material for: A qualitative study on factors influencing health workers’ uptake of a pilot surgical antibiotic prophylaxis stewardship programme in selected Georgian hospitals
Source: PLOS Glob Public Health. 2025 Apr 16;5(4):e0003493. doi: 10.1371/journal.pgph.0003493 (PMC12002478; doi:10.1371/journal.pgph.0003493)
Supplement: Attachment — Submitted filename: Reviewers comments.docx [file pgph.0003493.s003.docx]

**Title of the study:**
*A qualitative study on factors influencing health workers’ uptake of a pilot surgical antibiotic prophylaxis stewardship programme in selected Georgian hospitals*

**General Comments:**
The manuscript investigates the behavioral determinants influencing health workers' uptake of a pilot surgical antibiotic prophylaxis (SAP) stewardship program in selected Georgian hospitals. The manuscript is well-written, with an explicit methodology and comprehensive results. However, there are several points that require revision:

**Introduction**

- **Lines 61–63:** Recent data on antimicrobial resistance (AMR) should be incorporated to enhance the context specificity of the introduction. For instance, the following reference can be considered: https://doi.org/10.1016/S0140-6736(24)01867-1.
- **Lines 68–70:** The sentence, “Addressing surgical antibiotic prophylaxis (SAP) requires an appreciation and understanding of the behavioural and cultural context influencing health-care professionals’ practices and decisions,” could be revised. The term "addressing" may not be appropriate here as SAP is a type of antibiotic indication rather than a problem. Consider rephrasing this for clarity and precision.
- **Lines 80–82:** The authors discuss the inappropriate use of antibiotics, including in surgical prophylaxis, but there is no reference specifically addressing inappropriate use in surgical prophylaxis. If available, such a reference should be included to strengthen the argument.

**Methods**

- The methodology is well-articulated and thorough.
- **Figure 1:** The COM-B model presented in the figure could be tailored more specifically to ASP rather than focusing broadly on AMR and ABR. This adjustment would enhance its relevance to the study.
- **Lines 155–156:** The sentence, “Given that only one participant in each target group was engaged in the ASP at each hospital, data was almost entirely collected via semi-structured, in-depth interviews (IDIs),” could be clearer. Currently, it suggests that only one individual from each target group per hospital was involved in the ASP. Rewriting this for clarity would improve reader understanding.

**Results**

- The findings from the secondary target group (influencers) are not discussed. These participants likely have relevant perspectives or interests regarding the ASP program. Including their views would provide a more comprehensive analysis of the program's impact.

**Discussion**

- **Line 368:** If “Barriers to health worker ASP uptake related to the ASP delivery and health system context” is a heading, it should be formatted in bold. If it is part of the text, the sentence is incomplete as written.
- **Lines 371–373:** The discussion mentions the surgical site infection (SSI) reporting and feedback patterns of hospitals where the ASP was not introduced. If data on the reporting patterns of hospitals where the ASP was introduced is available, adding a comparative analysis would strengthen the discussion and provide valuable insights.
