# Supplementary material for: A qualitative study on factors influencing health workers’ uptake of a pilot surgical antibiotic prophylaxis stewardship programme in selected Georgian hospitals
Source: PLOS Glob Public Health. 2025 Apr 16;5(4):e0003493. doi: 10.1371/journal.pgph.0003493 (PMC12002478; doi:10.1371/journal.pgph.0003493)
Supplement: Attachment — Submitted filename: 01. Response to Reviewers.docx [file pgph.0003493.s005.docx]

**Response to Reviewers**

*3 December, 2024*

Dear Dr Vrinda Nampoothiri,

Thank you for your email and to the reviewers for taking time to provide such clear comments and suggestions. We have gone the reviewers’ comments and edited the manuscript accordingly. Please find below in blue the responses to the issues raised by each reviewer.

In accordance with the journal requirements outlined in your email, we have also:

- Completed and uploaded PLOS’ questionnaire on inclusivity in global research,
- Emailed full and correct funding information for the study,
- Uploading a Supplementary Information file on the findings data analaysed to inform the article, and
- Added an Author Summary as requested on lines 60-75

Please note, on lines 447-448 we have added the following to the acknowledgements section that was missed in the previous draft: “Ute Wolff Sönksen (Statens Serum Institut Denmark) for support in initial planning of the research”

Thank you again for your support and kind regards,

Dr. Sahil K. Warsi

Consultant, Antimicrobial Resistance, WHO Regional Office for Europe

**Response: Reviewer 1**

Could you explicitly list and discuss the key factors that were found to influence health workers' uptake of the surgical antibiotic prophylaxis stewardship programme? It would be helpful to:

1. Clearly enumerate these factors, perhaps in a dedicated section or a table.
2. Provide a brief description of each factor.
3. Discuss how each factor positively or negatively impacts the uptake of the stewardship programme.
4. If possible, rank or prioritize these factors based on their perceived importance or impact.

Thank you to Reviewer 1 for raising this issue; it has allowed us to consider a clearer way to depict the findings on COM-B related barriers. Specifically related to the points raised:

1. The barriers identified for each COM-B factor, along with a description, have now been succinctly depicted in Table 2 added on line 231, and an extra sentence introducing the table on lines 227-229.
2. The descriptions of the COM-B factors are provided in Figure 1 as included in the original submission.
3. We considered this point and reread our discussion and believe that the Impacts of the barriers and drivers for each COM-B factor on ASP uptake are inter-related and are considered together the discussion section in light of global research.
4. Unfortunately, as a small qualitative study, it is not feasible to rank barriers or drivers as they are also different across some target groups. We do welcome this comment to take on in future planning for any evaluation of the ASP.

**Response: Reviewer 2**

We greatly appreciate Reviewer 2’s detailed comments, alerting us to several issues that have made the manuscript clearer and more robust. We’ve responded to each point below in blue.

*Introduction*

- Lines 61–63: Recent data on antimicrobial resistance (AMR) should be incorporated to enhance the context specificity of the introduction. For instance, the following reference can be considered: <https://doi.org/10.1016/S0140-6736(24)01867-1>.

Thank you for this suggestion. We have added text to the manuscript (lines 82-86) as follows:

“Bacterial AMR in the WHO European Region was associated with over half a million deaths in 2019, putting considerable pressure on hospitals. Annually, AMR-related infection treatments in the European Union (EU) and European Economic Area (EEA) cost patients over 9.5 million extra days in hospital, and higher health expenditure and reduced workforce productivity cost countries approximately €11.7 billion.”

We have added the following references currently entries 4-6 in the bibliography:

4.	Mestrovic T, Robles Aguilar G, Swetschinski LR, Ikuta KS, Gray AP, Davis Weaver N, et al. The burden of bacterial antimicrobial resistance in the WHO European region in 2019: a cross-country systematic analysis. Lancet Public Health [Internet]. 2022 Nov [cited 2024 Dec 10];7(11):e897–913. Available from: https://linkinghub.elsevier.com/retrieve/pii/S2468266722002250

5.	Organisation for Economic Co-operation and Development (OECD). Fighting Antimicrobial Resistance in EU and EEA countries [Internet]. OECD; 2023. Available from: https://www.oecd.org/ en/publications/fighting-antimicrobial-resistance-in-eu-and-eea-countries_fdb1629f-en.html

6.	Organisation for Economic Co-operation and Development (OECD), European Centre for Disease Control (ECDC). Antimicrobial Resistance: Tackling the Burden in the European Union [Internet]. OECD; 2019. Available from: https://www.oecd.org/en/publications/antimicrobial-resistance-tackling-the-burden-in-the-european-union_33cbfc1c-en.html

- Lines 68–70: The sentence, “Addressing surgical antibiotic prophylaxis (SAP) requires an appreciation and understanding of the behavioural and cultural context influencing health-care professionals’ practices and decisions,” could be revised. The term "addressing" may not be appropriate here as SAP is a type of antibiotic indication rather than a problem. Consider rephrasing this for clarity and precision.

Thank you for pointing this out. We have now rephrased “addressing” to “optimizing” as it is more suitable on line 90.

- Lines 80–82: The authors discuss the inappropriate use of antibiotics, including in surgical prophylaxis, but there is no reference specifically addressing inappropriate use in surgical prophylaxis. If available, such a reference should be included to strengthen the argument.

We very much appreciate being alerted to this as we originally did have references specific to SAP. We have now included them. A reference to an unpublished ICARS survey on SAP in Georgian in line 104, as well as to global research on consequences of inappropriate SAP prescribing (line 104-106). The text lines 102-106 now reads:

“In Georgia, as in other countries, the inappropriate use of antibiotics, including for surgical prophylaxis, is characterized by a high use of broad-spectrum antibiotics and prolonged dosing, as evidenced in a 2020 SAP assessment across several hospitals in Tbilisi.(11–14) Evidence shows that such inappropriate SAP practices can have serious consequence for patients undergoing surgical procedure, including increased morbidity and mortality.(15)”

The references added are currently entries 14-15 in the bibliography:

14.International Centre for Antimicrobial Resistance Solutions (ICARS). Project Summary: Optimising the use of antibiotics for Surgical Antibiotic Prophylaxis (SAP) [Internet]. ICARS; Available from: https://icars-global.org/wp-content/uploads/2024/02/Georgia_SAP_2024.pdf

15.Habteweld HA, Yimam M, Tsige AW, Wondmkun YT, Endalifer BL, Ayenew KD. Surgical site infection and antimicrobial prophylaxis prescribing profile, and its determinants among hospitalized patients in Northeast Ethiopia: a hospital based cross-sectional study. Sci Rep [Internet]. 2023 Sep 6 [cited 2024 Feb 19];13(1):14689. Available from: https://www.nature.com/articles/s41598-023-41834-7

*Methods*

The methodology is well-articulated and thorough.

- Figure 1: The COM-B model presented in the figure could be tailored more specifically to ASP rather than focusing broadly on AMR and ABR. This adjustment would enhance its relevance to the study.

Thank you for the suggestion and we appreciate that this would indeed increase relevance of the figure. Unfortunately, Figure 1 is taken directly from the TAP Quick Guide, published by the WHO and cannot be modified. We have, however, tried to clarify this fact in the preceding text on lines 144-145, which now reads:

“Fig. 1 below from the TAP Quick Guide illustrates how the COM-B factors are approached through the TAP process.”

- Lines 155–156: The sentence, “Given that only one participant in each target group was engaged in the ASP at each hospital, data was almost entirely collected via semi-structured, in-depth interviews (IDIs),” could be clearer. Currently, it suggests that only one individual from each target group per hospital was involved in the ASP. Rewriting this for clarity would improve reader understanding.

Thank you again for pointing this out. It is indeed the fact that only one person per target group was involved in the SAP in each hospital. This is because of the small size of the surgical teams. We have amended the sentence on line 179-180 to state this more clearly as follows:

“Only one participant in each target group was engaged in the ASP at each hospital, which was the entire surgical team at most hospitals, thus data was almost entirely collected via semi-structured, in-depth interviews (IDIs)”.

*Results*

- The findings from the secondary target group (influencers) are not discussed. These participants likely have relevant perspectives or interests regarding the ASP program. Including their views would provide a more comprehensive analysis of the program's impact.

We appreciate this point as raised. Unfortunately, the secondary target group interviewees did not discuss their own behaviour or evaded responses, but did talk about perceptions of primary target groups’ behaviours. These insights were considered and weighed as reported behavioural determinants in the analysis, as indicated by a reference to a professional association representative response on lines 268-271. We have also clarified this, by editing lines 218-222 as follows:

“Findings on factors affecting ASP uptake show similarities across primary target groups as well as differences between ASP-introduced and non-introduced hospitals. Secondary target group participants’ responses mainly captured factors affecting primary target groups’ behaviours, and were thus included in primary target group COM-B analysis.”

*Discussion*

- Line 368: If “Barriers to health worker ASP uptake related to the ASP delivery and health system context” is a heading, it should be formatted in bold. If it is part of the text, the sentence is incomplete as written.

The sentence was a statement, but we appreciate ‘related’ might be an unclear verb, and have edited the sentence on line 400 to:

“Barriers to health worker ASP uptake were connected to the ASP delivery and health system context.”

- Lines 371–373: The discussion mentions the surgical site infection (SSI) reporting and feedback patterns of hospitals where the ASP was not introduced. If data on the reporting patterns of hospitals where the ASP was introduced is available, adding a comparative analysis would strengthen the discussion and provide valuable insights.

Thank you for raising this, we think the text was maybe not entirely clear. The paragraph does not mention reporting and feedback patterns for ASP introduced and non-introduced hospitals, but it aims to show the ASP uptake can be connected to (a) the challenging introduction of the ASP, and also potentially impacted by (b) the context of no national level SSI reporting. We’ve attempted to clarify this on line 403-404 so that it now reads:

“Regarding health system context, the unavailability of national-level information on SSIs…”
