# Supplementary material for: A qualitative study on factors influencing health workers’ uptake of a pilot surgical antibiotic prophylaxis stewardship programme in selected Georgian hospitals
Source: PLOS Glob Public Health. 2025 Apr 16;5(4):e0003493. doi: 10.1371/journal.pgph.0003493 (PMC12002478; doi:10.1371/journal.pgph.0003493)
Supplement: Attachment — Submitted filename: 01. Response to Editor Comments.docx [file pgph.0003493.s006.docx]

**Response to Editor Comments**

*February 18, 2025*

Dear Dr Bipin Adhikari,

We are pleased to hear the reviewers accepted our response and welcome your additional comments. We have edited the manuscript accordingly and provided our responses to your questions below in blue. We have also attached an updated version of the manuscript with changes highlighted in yellow, as well as a clean version of the updated manuscript.

Thank you for your support and best regards,

Dr. Sahil K. Warsi

Consultant, Antimicrobial Resistance, WHO Regional Office for Europe

**Response to Editor**

- You have used COM-B behavioural model which is fine, but for readers, it would be important to inform #1 that there are several behavioural models, add a line on how they present similarities and differences; and #2 add rationale behind using this particular model.

Thank you for raising this, as indeed readers might not be aware of behavioural change models and it might not be clear why the COM-B is employed. We’ve updated the manuscript to highlight that the BCW is used in the TAP method to ensure a robust approach to intervention development, and the COM-B model relates to this framework. The existence of other models had already been mentioned with regard to the COM-B model, but we have further clarified what lends strength to the COM-B approach in considering public health behaviour. After the changes, lines 141-151 now read as follows:

To ensure a rigorous, theory-based approach to intervention development, the TAP method employs the BCW framework, which links specific sources of behaviour with discrete intervention functions and policy categories based on research evidence. Using this framework, the TAP method ensures developed interventions address relevant behavioural barriers and drivers with evidence-based approaches. The COM-B theory at the core of the BCW framework is built on a review of 19 existing behaviour change frameworks, each of which approaches public health behaviour from various perspectives, and presents a model for exploring overlapping and interacting individual and contextual factors that affect individuals’ performance of public health behaviours. The COM-B theory holds that public health behaviour change is influenced by the interlinked factors of individuals’ capability, opportunity, and motivation to enact specific behaviours.

- Page 15, Line 293 to 310

I see the challenge arising when patients (or their family members) take OTC antimicrobials. This is quite inductive based on your data and I recommend authors to discuss this challenge in the discussion, address why people take OTC antimicrobials, compare the Georgian context with the literature that may explain. This could be outside the remit of intended receipients of training but still likely to affect the intervention. Thus this warrants a discussion in few lines.

We appreciate the invitation to clarify the context around OTC sales, which is important to also drive home the importance of effective health worker communication. We have added this context to the discussion and lines 419-430 have been edited as follows:

Another factor cited as affecting long-term ASP sustainability was patients’ demand for and access to antibiotics OTC, leading to their disregard of doctors’ treatment advice. OTC antibiotics demand is affected by multiple factors including AMR or antibiotics knowledge gaps, individual convenience and cost considerations, health provider AMR awareness, and policy and guideline enforcement.(32,33) Research in Georgia, as in other countries in the region, shows OTC antibiotics demand is linked to patient convenience in wanting to save time and money on doctor appointments or buying drugs from more expensive pharmacies rather than cheaper community pharmacies, as well as knowledge gaps leading to over confidence in self-prescribing.(34,35) Public awareness of AMR is a widespread issue in the country, and study nurses did report their ASP training equipped them with knowledge and skills to effectively communicate with patients on AMR and appropriate antibiotic use, which administrators indicated was a particular challenge.

Based on the above changes the following citaitons have were added to the manuscript:

32.	Adhikari B, Pokharel S, Raut S, Adhikari J, Thapa S, Paudel K, et al. Why do people purchase antibiotics over-the-counter? A qualitative study with patients, clinicians and dispensers in central, eastern and western Nepal. BMJ Glob Health [Internet]. 2021 May [cited 2025 Feb 18];6(5):e005829. Available from: https://gh.bmj.com/lookup/doi/10.1136/bmjgh-2021-005829

33.	Belachew SA, Hall L, Erku DA, Selvey LA. No prescription? No problem: drivers of non-prescribed sale of antibiotics among community drug retail outlets in low and middle income countries: a systematic review of qualitative studies. BMC Public Health [Internet]. 2021 Dec [cited 2025 Feb 18];21(1):1056. Available from: https://bmcpublichealth.biomedcentral.com/articles/10.1186/s12889-021-11163-3

34.	Kaae S, Ghazaryan L, Pagava K, Korinteli I, Makalkina L, Zhetimkarinova G, et al. The antibiotic knowledge, attitudes and behaviors of patients, doctors and pharmacists in the WHO Eastern European region – a qualitative, comparative analysis of the culture of antibiotic use in Armenia, Georgia, Kazakhstan, Moldova, Russia and Tajikistan. Res Soc Adm Pharm [Internet]. 2019 May 23; Available from: http://www.sciencedirect.com/science/article/pii/S1551741118308416

35.	Rachina S, Zakharenkova P, Kozlov R, Palagin I, Mamchich D, Strelkova D. The Antibiotic Knowledge, Attitudes, and Behaviors of Patients Purchasing Antibiotics without Prescription: Results of National Survey. Duradoni M, editor. Adv Public Health [Internet]. 2023 Nov 25 [cited 2025 Feb 18];2023:1–11. Available from: https://www.hindawi.com/journals/aph/2023/3306067/
